# The PhINEST study – Pharyngeal ICU Novel Electrical Stimulation Therapy: Study protocol of a prospective, multi-site, randomized, sham-controlled, single-blind (outcome assessor-blinded) study

**DOI:** 10.1097/MD.0000000000019503

**Published:** 2020-03-13

**Authors:** Joerg C. Schefold, Minna Bäcklund, Tero Ala-Kokko, Patrick Zuercher, Rajat Mukherjee, Satish Mistry, Stephan A. Mayer, Rainer Dziewas, Jan Bakker, Stephan M. Jakob

**Affiliations:** a Department of Intensive Care Medicine, Inselspital, Bern University Hospital, University of Bern, Switzerland; b Department of Intensive Care Medicine, Meilahti Tower Hospital, Helsinki University Hospital; c Department of Intensive Care Medicine, Oulu University Hospital, Finland; d Cytel Inc., 675 Massachusetts Avenue, Cambridge, MA; e Department for Clinical Research, Phagenesis Limited, Manchester Science Park, Manchester, United Kingdom; f Department of Neurology, Henry Ford Health System, Detroit, MI; g Department of Neurology, University Hospital Münster, Münster, Germany; h Department Pulmonology and Critical Care. NYU Langone and Columbia University Medical Center New York, USA and Department Intensive Care Adults, Erasmus MC University Medical Center, Rotterdam, The Netherlands.

**Keywords:** FEES, intensive care unit, mechanical ventilation, oropharyngeal dysphagia, PES, pharyngeal electrical stimulation

## Abstract

**Introduction:**

Post-extubation dysphagia is commonly observed in ICU patients and associated with increased aspiration rates, delayed resumption of oral intake/ malnutrition, prolonged ICU and hospital length of stay, decreased quality of life, and increased mortality. Conventional therapeutic approaches are limited. Pharyngeal electrical stimulation (PES) was previously shown to improve swallowing function and airway safety in severely dysphagic tracheostomised stroke patients.

**Methods:**

In a multi-center, single-blind, 1:1 randomized controlled study, up to 400 (360 evaluable) mixed emergency adult ICU patients with recent extubation following mechanical ventilation and confirmed oropharyngeal dysphagia will be enrolled at investigational academic ICUs. Primary objective is to evaluate the effectiveness of PES in reducing the severity of unsafe swallows. Patients will be randomized to receive PES (or sham) treatment on 3 consecutive days in addition to best supportive care. Primary endpoint is a composite of 2 endpoints with hierarchy based on clinical priorities:

1) Swallowing safety based on worst penetration-aspiration-scale (PAS) score in series of up to 4 boli using thin stimuli approx. From 24 to 60 hours after treatment completion, converted to a trichotomized ordinal response of safe (PAS 1–3), penetration (PAS 4–5), or aspiration (PAS 6–8).
2) Dysphagia Outcome and Severity Scale scores determined by bedside assessment 7 ± 1 days after treatment completion. Oropharyngeal dysphagia will be assessed by Fiberoptic Endoscopic Evaluation of Swallowing by blinded study staff. Patients will be followed-up for a maximum of 90 days.

**Discussion:**

This study will evaluate the effects of PES on swallowing safety in critically ill ICU patients post mechanical ventilation with oropharyngeal dysphagia.

## 1 Introduction

Oropharyngeal dysphagia, also referred to as dysphagia or “disordered swallowing”, is an abnormality of the swallow physiology (reviewed in^[1]^). It is observed frequently in patients with stroke^[2]^ and additional neurological conditions, head and/or neck cancer,^[3]^ cervical spine surgery,^[4]^ prolonged intubation,^[5,6]^ tracheostomy,^[7,8]^ and/or mechanical ventilation.^[9]^ Although the literature reports a high incidence of dysphagia following endotracheal intubation for more than 48 hours, the reported incidence rate varies widely from to 3% to 83%.^[5,10,11]^ Studies have also shown that prolonged intubation might be a risk factor for dysphagia,^[12,13]^ as artificial airways increase the risk of upper airway injury and concomitant laryngeal pathologies.^[14–16]^ However, causes for “ICU-acquired” dysphagia are multifactorial and with many risk factors still currently unknown.^[1]^

Recently, the incidence rates of post-extubation dysphagia in a large cohort of 933 mixed medical and surgical ICU patients was reported by us in the DYnAMICS study.^[9]^ We revealed the significance of post-extubation dysphagia in the ICU with 12.4% (n = 116/933) of the total ICU population (18.3% of emergency and 4.9% of elective ICU patients) affected. Moreover, 60% of patients leaving the ICU having been identified as dysphagia positive, were still dysphagic at hospital discharge. Furthermore, the presence of dysphagia was associated with increased morbidity (e.g., increased resource use, increased hospital length of stay) and, after adjustment, dysphagia remained an independent predictor for 28-day and 90-day mortality (excess 90-day mortality 9.2%).^[9]^ Diagnosis of post-extubation dysphagia is most commonly established by ICU nurses at the bedside (dysphagia screening) followed by an assessment by a trained speech and language pathologist (SLP) or occupational/ physio-therapist.^[1,17,18]^ Fiberoptic Endoscopic Evaluation of Swallowing (FEES) is an instrumental examination technique that allows visualization of the pharynx during swallowing. Data show that FEES is well tolerated and considered a safe technique^[19–22]^ with minimal complications.^[23]^

Treatment for dysphagia is typically limited to dietary adjustment and postural changes as well as compensatory manoeuvres aiming to improve swallowing function.^[1,17,24]^ The therapeutic efficiency of such ‘traditional’ therapies is limited, with no evidence for clinical benefit in ICU patients.^[1,25]^ Novel interventional approaches to treat dysphagia are therefore warranted. Pharyngeal electrical stimulation (PES) is a novel technique that offers a new treatment option for dysphagic patients and in 2 recent studies, the benefits of PES in severely dysphagic tracheotomized stroke patients^[26,27]^ were demonstrated. In the first study, Suntrup et al^[27]^ conducted a single-center, randomized controlled pilot study in 30 severely dysphagic tracheotomized acute stroke patients. Patients were given PES or sham and thereafter assessed for decannulation readiness using a FEES-based decannulation algorithm.^[28]^ In their study, Suntrup et al^[27]^ found that dysphagia improved enabling decannulation in 15/20 (75%) patients of the treatment group, whereas only 2/10 (20%) of control patients showed spontaneous remission of post-stroke dysphagia sufficient enough to allow for subsequent removal of the tracheal cannula. In the follow-on multi-center, randomized controlled PHAST-TRAC study by Dziewas et al^[26]^ conducted in 69 severely dysphagic tracheotomized stroke, PES was associated with 17/35 (49%) patients being ready for tracheostomy decannulation when compared to just 3/34 (3%) controls. PES is considered to stimulate afferent sensory feedback resulting in enhanced reorganization of the swallow-related motor-cortex, faciliatory activation of cortico-bulbar pathways, and increased salivary substance P levels, a swallow-related neurotransmitter, inducing more quantitative and qualitative swallows.^[1,29–31]^ Moreover, the relationship between PES treatment efficacy and short times to treatment (favoring treatment earlier after stroke, or with a shorter duration of mechanical ventilation) in the PHAST-TRAC study^[26]^ are thought to be related to the development of critical illness dysphagia due to critical illness polyneuropathy and myopathy in patients with prolonged ICU treatment and mechanical ventilation.

We embarked to design a clinical study that evaluates the effectiveness of PES in reducing the severity of unsafe swallows in mixed emergency adult ICU patients with recent extubation following mechanical ventilation.

## 2 Objectives

### 2.1 Primary objective

To evaluate the effectiveness of PES (Phagenyx) treatment in reducing the severity of unsafe swallows.

### 2.2 Secondary objectives

- To evaluate and further characterize the effectiveness of PES treatment in improving nutritional management and reducing the severity of dysphagia.
- To evaluate the effectiveness of PES treatment on general patient health outcomes.

### 2.3 Other objectives and supplementary data collected

Exploratory additional analysis of the effectiveness of PES treatment in reducing dysphagia severity and other health outcome measures.

## 3 Methods

### 3.1 Design

**Figure 1:**
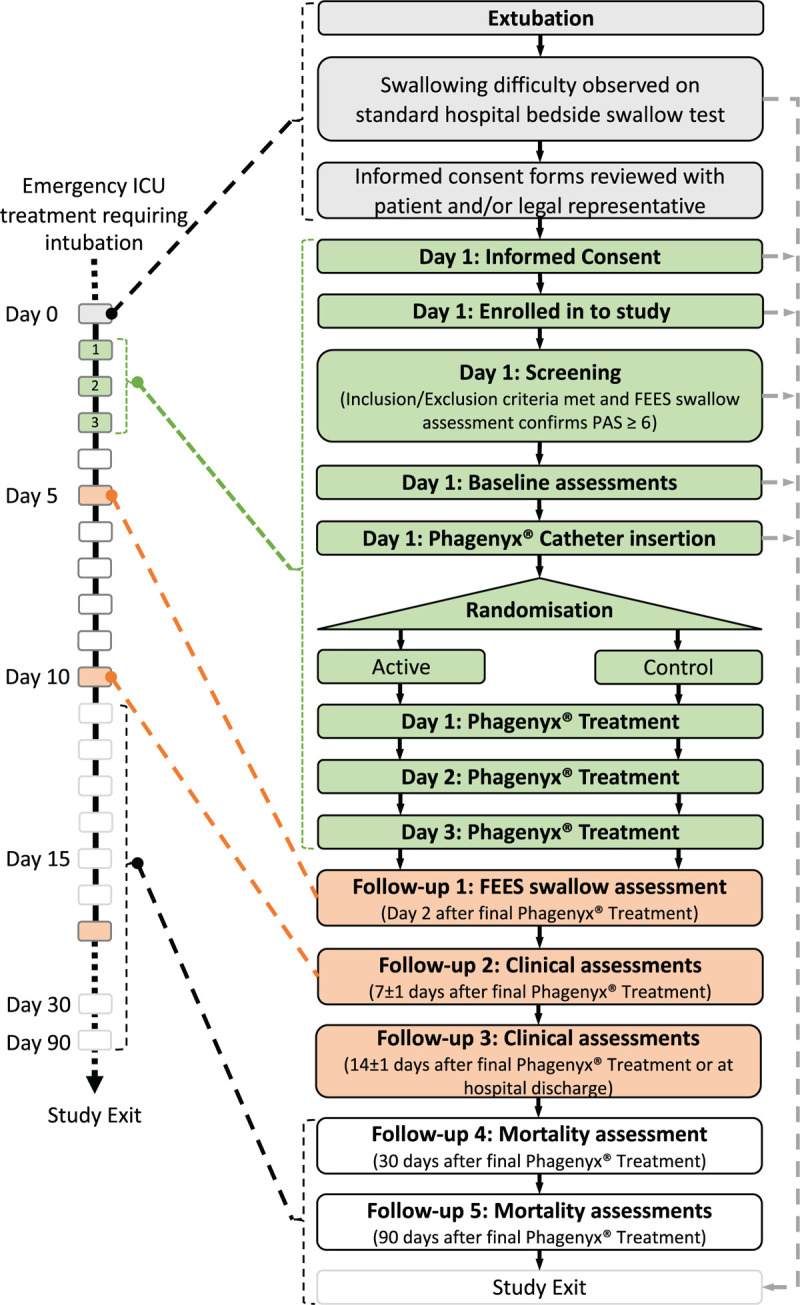
Study flowchart.

Prospective, multi-center, 1:1 randomized, sham-controlled, patient-masked, outcome assessor-blinded study designed to assess the effects of PES for the treatment of oropharyngeal dysphagia after invasive mechanical ventilation (of any length of time; by means of naso- or oro-tracheal tube) in critically ill intensive care unit (ICU) patients (active study protocol version 1.0, dated Nov 8, 2018). A study flowchart is provided (Fig. 1). Patient randomization will occur after dysphagia severity is classified as ‘aspiration’ (PAS score ≥ 6) on FEES assessment, and the Phagenyx Catheter is successfully placed. Randomization will be on a 1:1 basis at each site stratified for neurological vs non-neurological reason for admission (APACHE IV diagnostic group). All randomized patients will receive either PES treatment or sham (comparator) by a health care professional who is unblinded to the treatment assignment (PES treater). Sham treatment will be performed using the same device without delivering energy. Administration of all protocol-specific assessments, other than PES or sham, will be conducted by study personnel who are blinded to the treatment assignment, including therapists delivering standard dysphagia care (other forms of electrical stimulation treatment for dysphagia are not permitted in this study). FEES assessments of swallowing safety and decisions on patient treatment will be made by the local clinical team responsible for the patient's care. Study data will be collected using an electronic Case Report Form (eCRF) (SYNCRONY, Syntactx Technologies LLC, NY).

An Independent FEES Review Committee (IFRC), external to the study sites, will be established for analysis of all study-related FEES assessment parameters and to confirm/validate the PAS scoring of the local clinical FEES assessor(s). FEES results will not be scored in duplicate but discrepancies in ratings between the local team and IFRC, or concerns about the quality of submitted records will be reviewed and discussed with the local team for (re-)training purposes only. All FEES records will be coded so that records are only identified by site number, bolus number, patient number, and study visit (i.e., screening or follow-up). The IFRC will be blinded to the treatment assignment as well as the actual treatment received.

Six or more investigational academic hospital sites across Europe are anticipated to participate in this study. Factors considered for site qualification of ICUs include, but are not limited to: potential number of suitable patients, availability, and experience of conducting FEES. The enrolment period is expected to be approximately 48 months from the time of first patient recruitment to the final study visit. Each patient's participation will last for up to 90 days with assessments at the following intervals: screening, baseline, day 2 (approx. 24–60 hours), 7 ± 1 days, 14 ± 1 days or hospital discharge (whichever is first) after completion of the final PES treatment. 30-day and 90-day mortality data will also be collected from the patients (electronic health record) medical notes - this will not require an additional visit but a phone call may be conducted depending on the patient's status. Dysphagic patients enrolled into the study will continue to receive nutrition as deemed appropriate by the ICU team. Patients with a nasogastric feeding tube in place at the time of screening/enrolment will have their nasogastric tube replaced with the Phagenyx Catheter since the Phagenyx Catheter may also be used to deliver nutrition to the patient as required for up to 30 days after placement.

## 4 Study population

Up to 400 (360 evaluable) mixed emergency (unplanned) admission adult critically ill ICU patients post invasive mechanical ventilation with confirmed oropharyngeal dysphagia will be recruited in to this study.

## 5 Inclusion and exclusion criteria

Pre-screening inclusion criteria (all of the following criteria must apply):

- Age ≥18 and ≤85 years,
- Emergency ICU admission (unplanned admission),
- Recent extubation following invasive mechanical ventilation (of any duration) by means of endotracheal tube,
- Presence of post-extubation dysphagia as determined by the participating sites’ standard of care (SOC).

Screening / Enrolment Criteria: To be eligible for enrolment in this study, an individual must meet all of the following additional criteria:

- Presence of written informed consent according to respective national guidelines (patients are considered enrolled once informed consent is provided),
- Dysphagia severity status classified according to PAS on FEES assessment as ‘aspiration’ (PAS score ≥6),
- Richmond Agitation and Sedation Scale (RASS) score within the range of −1 to +2 *(i.e., if score equals* −*2,* −*3,* −*4 or +3, +4, patient is excluded)*,

Randomization inclusion criteria (post consent): The following additional criteria must be met for randomization:

- Successful placement and subjective tolerance of the Phagenyx Catheter within 2 days of extubation.

Exclusion criteria: (any of the following):

- Nasal anatomical deformity, nasal airway obstruction; patient who have had recent oral, nasal or oesophageal surgery or patient presenting with facial and/or cranial fractures or any other circumstance (e.g., history of oesophageal perforation, stricture, pouch, resection or rupture) where placement of a standard nasogastric feeding tube would be deemed unsafe,
- Cardiac or respiratory condition that might render the insertion (placement) of a catheter into the throat unsafe,
- Presence of a permanently implanted electrical device
- Are pregnant (pregnancy test) or known lactating women,
- Have non-neurogenic dysphagia (e.g., Cancer-related),
- Any prior tracheostomy,
- Patients who at the time of extubation have a treatment limitation, life expectancy, or are moribund, that prevents or would prevent compliance with study-specific instructions or procedures (as judged by the investigator)
- Severe cognitive impairment or other reasons that prevents compliance with study-specific instructions or procedures (as judged by the investigator),
- Previous history of dysphagia of any origin,
- Pre-existing tube feeding of any form (e.g., percutaneous gastric/enteral feeding tube related to previous injuries indicating previous dysphagia. Nasogastric feeding tubes are not an exclusion criterion),
- Participation in another interventional study (medicinal or device) that could influence the outcomes of PES,
- Treatment of dysphagia with other forms of electrical stimulation.

## 6 Outcome measures

### 6.1 Primary endpoint

A composite endpoint using the Finkelstein-Schoenfeld (FS)^[32]^ (win-ratio) statistic, (see statistical considerations) analyzed on a hierarchy, based on clinical priorities, of the following 2 endpoints:

- Swallowing safety based on worst PAS score in a series of up to 4 boli
  o using thin stimuli (water) for each patient as determined by a FEES assessment on day 2 (approx. 24–60 hours) after completion of the final PES treatment, converted to a trichotomized ordinal response of safe (PAS 1–3), penetration (PAS 4–5), or aspiration (PAS 6–8),
- Dysphagia Outcome and Severity Scale (DOSS) score determined by bedside assessment 7 ± 1 days after completion of the final PES treatment.

### 6.2 Secondary endpoints

1. Changes in nutritional management and severity of dysphagia will be assessed by:
  - Dysphagia status as measured by DOSS at baseline, day 2 (approx. 24–60 hours), 7 ± 1 days and at 14 ± 1 days (or hospital discharge if earlier) after completion of the final PES treatment (to assess changes in functional severity of dysphagia over time),
  - Time (days) from randomization to removal of feeding tube,
  - Time (days) to first oral intake (if applicable. Oral intake is minimally defined as movement from for example FOIS 3 (Tube dependent with consistent intake of liquid or food) to FOIS 4 (Total oral diet of a single consistency) or from DOSS 2 (Moderately severe dysphagia) to DOSS 3 (Moderate dysphagia),
  - Total days of enteral feeding,
  - FOIS scale at baseline, day 2 (approx. 24–60 hours), 7 ± 1 days and at 14 ± 1 days (or hospital discharge if earlier) after completion of the final PES treatment.
  - General health outcomes will be assessed by:
  - Time (days) from extubation to ICU discharge,
  - ICU LOS (Length of stay in ICU),
  - Hospital LOS (Length of stay in hospital)
  - Number of patients with re-intubation during hospital stay
  - Mortality.

### 6.3 Other endpoints and supplementary data collected

Additional data for exploratory analysis will be collected in relation to dysphagia severity and general patient outcomes. For changes in severity of dysphagia between FEES assessments, the IFRC will evaluate secretion severity (Murray Secretion Severity Rating Scale (SSS)), bolus residue (Yale Pharyngeal Residue Severity Rating Scale (YRS)) and global dysphagia severity (Global Dysphagia severity Score (GDS). Further, swallowing safety and efficiency will be analyzed using the worst PAS score for each bolus consistency for each patient as determined by a FEES assessment on day 2 (approx. 24–60 hours) after completion of final PES treatment, converted to a dichotomized ordinal response of non-aspirator (PAS 1–5) or aspirator (PAS 6–8) (performed to address presence of aspiration, a key medical problem). General patient health outcome data collected and to be analyzed include time from extubation to hospital discharge (days), days on antimicrobials post-extubation (while in ICU), hospital discharge destination (home, home + care, nursing home, other hospital, rehabilitation center), ICU readmission rate during hospital stay, number of and reason for tracheostomies per group after PES treatment during hospital stay and total number of chest X-rays during hospital stay after ICU discharge for suspected pneumonia. Phagenyx treatment parameters (catheter insertion-related, ease of use, threshold, tolerance and stimulation levels) and nursing workload (TISS-28) data will also be collected.

## 7 Study intervention and comparator

For the study intervention (active PES) and sham (comparator), a commercial device (Phagenyx, Phagenesis Ltd, Manchester, UK), which comprises a nasogastric feeding catheter housing stimulation ring-electrodes, and a computerized base station that delivers stimulation in the range 1 to 50 mA at 5 Hz will be used. All eligible patients will undergo PES catheter placement. Appropriate adjustment and placement of the PES catheter in each patient is ensured using catheter markings, aspirate test or X-ray, and fixation of the catheter using a securing clamp. Catheter ease of use will be documented in the eCRF. PES treatment (active) and sham (comparator) will be delivered for 10 minutes per day (as per the device's programming and CE) for 3 consecutive days^[33,34]^ after randomization and performed by a health care professional (nurse, therapist, or physician) unblinded to treatment assignment (PES treater). Sham treatment will be performed using the same device without delivering any energy. To deliver (active) PES, the current intensity (mA) at which PES-treatment will be delivered is individually adjusted and optimized at the start of each treatment session by the PES treater in response to patient responses. This treatment optimization procedure involves increasing the current intensity incrementally from 1 mA to detect the perceptual threshold (PT – patient first aware of stimulation) and then to a maximum tolerated threshold (MTT – patient no longer wants current increased further) intensity levels three times each respectively. Thereafter, the optimal treatment intensity is automatically calculated by the base station using the average values of the three trials according to the formula PT + 0.75 × (MTT − PT).^[26,35]^

To deliver sham PES, the optimization procedure is imitated exactly as in (active) PES to mitigate any bias or effect of time spent interacting with the patient during (active) PES, however, no current is applied. Treatment parameters will be documented in the eCRF.

## 8 Randomization

Patients will be randomly allocated to PES treatment or sham (comparator) according to a permutated block randomization stratified by site and reason for ICU admission (neurological vs non-neurological according to APACHE IV diagnostic criteria) using a random mixture of blocks of size 2 and 4. The randomization ratio will be 1:1. The PES treater(s) at site shall not be blinded to the patient's randomization assignment in order to be able to deliver PES treatment or sham (comparator). Randomization assignment will be via the web-based study EDC with access restricted to authorized PES treaters. The randomization page of the EDC will only become available once the patient is confirmed to have met all of the inclusion/exclusion criteria and the treatment catheter is successfully placed and tolerated.

## 9 Procedures for emergency unblinding

In the event of a medical emergency which requires identification of an individual patient's randomization and (PES or sham) treatment information, investigators will be able to access this information via the PES treater(s). Reasons for unblinding will be documented in the patient's medical records.

## 10 Study assessments

### 10.1 Screening and baseline assessments

**Figure 2:**
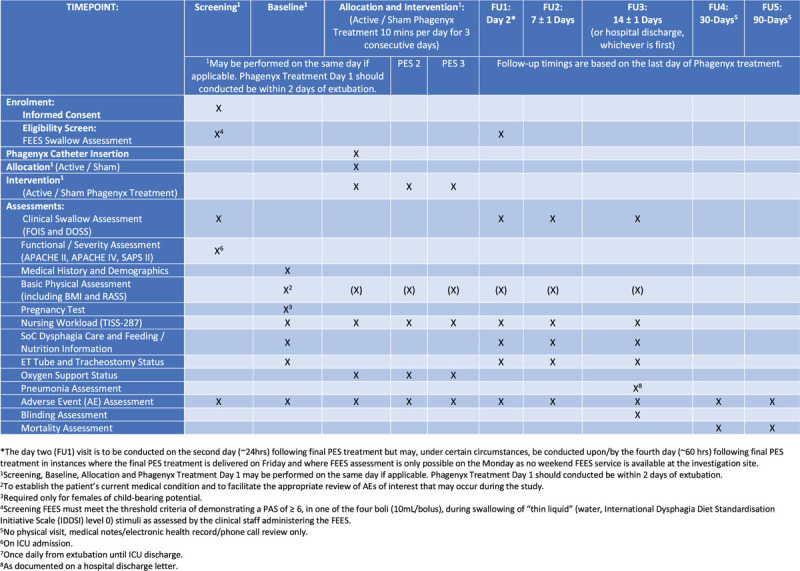
SPIRIT Figure.

Informed consent will be obtained at the screening visit, by the local principal investigator (or designee) from the patient or their legal representative according to respective local/national guidelines using approved consent forms (study flowchart Fig. 1). A SPIRIT figure is provided (Fig. 2).

In addition to the assessments detailed below, a medical history, demographic data and the patient's current medical status will be recorded at the baseline visit. Medical history and demographic data will be obtained from the patient's medical record. Other information captured will include: functional severity data (e.g., APACHE II, SAPS II), BMI, RASS, nursing workload (TISS-28), standard of care dysphagia care and feeding / nutrition being provided to the patient, ET tube and tracheostomy status. Information obtained from these assessments will be recorded in the eCRF.

### 10.2 Fiberoptic endoscopic evaluation of swallowing (FEES)

FEES is recognized, validated, and routinely used in many hospitals across Europe for instrumental dysphagia assessment. It is considered a gold standard technique for airway- and swallow-safety^[19–22]^ assessment and is the recommended methodology in current guidelines.^[20,36]^ Complications such as discomfort, gagging, vomiting, vaso-vagal syncope, anterior or posterior epistaxis or rare complications such as laryngospasm are expected to occur in < 1% of examinations.^[23]^ In this study, FEES will be performed via nasal induction of an endoscope into the pharynx with visualization of the larynx (details are given elsewhere ^[1]^). FEES assessments will be conducted at screening and on day 2 (approx. 24–60 hours) after completion of the final PES treatment. The FEES assessment will capture:

- 1 × 30 second video prior to any boli stimuli to assess secretions severity.
- 4 × 10 ml swallow trials with thin liquid (water, International Dysphagia Diet Standardisation Initiative (IDDSI ^[37]^) level 0) stimuli.
- 2 × 10 ml swallow trials with semi-solid (puree, IDDSI level 4) stimuli.
- 2 × non-obligatory soft and bite-sized swallow trials with solid (white bread without the crust approximately 1.5 cm × 1.5 cm × 0.5 cm, IDDSI level 6) stimuli presented only to patients in whom no residues are observed on thin liquid and puree swallow trials.

Data will be entered in the eCRF. Coded video records will also be provided to the IFRC for further analysis and validation of local PAS scores for the thin liquid stimuli (for (re)training purposes only). IFRC members will score videos blinded to the site and other member's scores to allow unbiased scoring. FEES results will not be scored in duplicate, PAS ratings by the local site investigator will be used for all primary endpoint analyses and clinical decision-making.

### 10.3 FEES stopping criteria

If gross aspiration (PAS ≥6, see below) of two consecutive bolus trials of the same consistency are observed, that consistency should be stopped and the investigator administering the FEES (or trained and authorized designee) should decide either to move to the next consistency (to assess any potential dietary modification) or terminate the FEES. A ‘Stop’ may be applied at any time if the investigator administering the FEES (or trained and authorized designee) feels it to be unsafe for the patient to continue.

## 11 Assessment scoring scales

### 11.1 Penetration aspiration scale score (incl. definitions):

The PAS is a validated 8-point ordinal scale that quantifies penetration and aspiration events observed during instrumental swallow assessment.^[38]^ The scale ranges from 1 (normal swallow: material does not enter airway) to 8 (severe aspiration: material enters the airway, passes below the vocal folds & no effort is made to eject). Swallowing severity will be assessed using the PAS observed during FEES at screening and on day 2 (approx. 24–60 hours) after completion of the final PES treatment. The worst PAS score for each thin liquid bolus will be recorded in the eCRF. IFRC PAS scores will be entered in a separate portion of the eCRF.

### 11.2 Functional oral intake scale (FOIS)

The FOIS was developed to document the functional level of oral intake of food and liquid in stroke patients with dysphagia.^[39]^ It is a 7-point ordinal scale easily completed by clinicians based on information contained in medical charts, dietary journals, and/or patient reports. Verification of patient reports may be obtained by a spouse or family members or from a variety of sources for institutionalized patients. The score ranges from 1 (nothing by mouth) to 7 (total oral diet with no restriction). FOIS scores will be recorded in the eCRF at the screening, day 2, 7 ± 1 days and 14 ± 1 days visits.

### 11.3 Dysphagia outcome and severity scale (DOSS)

The DOSS is a simple, easy-to-use, 7-point scale developed to systematically rate the functional severity of dysphagia based on objective assessment and make recommendations for diet level, independence level, and type of nutrition.^[40]^ The score ranges from 1 (severe dysphagia) to 7 (normal in all situations). DOSS scores will be recorded in the eCRF at the screening, day 2, 7 ± 1 days and 14 ± 1 days visits.

### 11.4 Murray secretion severity rating scale (SSS)

The SSS is a simple, easy-to-use, 4-point scale developed to systematically rate the graduation of accumulated secretions within the pharyngolarynx and trachea during an objective assessment using FEES^[41]^ by trained clinicians. The score ranges from 0 (normal) to 3 (severe). Secretion severity will be assessed by the IFRC using the SSS observed during FEES at screening and on day 2 (approx. 24–60 hours) after completion of the final PES treatment. SSS scores will be recorded in the eCRF.

### 11.5 Yale pharyngeal residue severity rating scale (YRS)

The Yale Pharyngeal Residue Severity Rating Scale (YRS) is a reliable, validated, anatomically defined, and image-based five-point ordinal rating scale to determine severity of post-swallow pharyngeal residue location (vallecula and pyriform sinus) and amount (none, trace, mild, moderate, and severe) during FEES.^[42]^ Residue severity will be assessed by the IFRC using the YRS observed during FEES at screening and on day 2 (approx. 24–60 hours) after completion of the final PES treatment. YRS scores will be recorded in the eCRF.

### 11.6 Global dysphagia severity rating scale (GDS)

The Global Dysphagia Severity ratings scale (GDS) is a simple, easy-to-use, 4-point scale developed to systematically rate the severity of dysphagia during an objective assessment using FEES^[43]^ by trained clinicians. Dysphagia severity will be assessed by the IFRC using the GDS observed during FEES at screening and on day 2 (approx. 24–60 hours) after completion of the final PES treatment. GDS scores will be recorded in the eCRF.

## 12 Data collection, management, and confidentiality

Study data will be collected and stored in a validated, password protected electronic data capture (EDC) system using eCRFs. Designated site personnel will be trained to enter data into the web-based, study-specific EDC system. The EDC system allows for tracking of all data elements and any changes made (full audit trail).

As per ISO14155: 2011 (section 6.3, 6.7), in order to assure appropriate conduct of the study, participating investigators and/or institutions shall provide direct access to source data during and after the clinical investigation for monitoring, audits, EC review and regulatory inspections. Patient confidentiality will be strictly held in trust by the investigators, study staff, the sponsor(s) and their agents to the extent allowed by law, including Personally Identifiable Information. Investigators will ensure protection of patient personal data and that all reports, publications, and any other disclosures, except where required by law are identified only by the patient identification number and site identification number to maintain patient confidentiality. All patient study records will be kept safely in an access-controlled area. At the end of the study, all records will continue to be kept in a secure location for at least 15 years (or per local regulations) after the study is closed.

## 13 Clinical monitoring, quality assurance and quality control

Clinical site monitoring will be conducted to help ensure that the rights and well-being of patients are protected, that the reported study data are accurate, complete, verifiable and that the conduct of the study is compliant with the currently approved protocol/amendment(s) and with applicable regulatory requirement(s) including ISO14155: 2011 and ICH GCPs. The sponsor or designee Clinical Research Organization (Syntactx EU Limited) will train all Investigators and relevant study staff at or prior to site initiation and monitor the study throughout its duration. Study monitors will visit each site at appropriate intervals to review clinical data for accuracy and completeness and to help ensure compliance with the protocol. A study termination (close-out) monitoring visit will be conducted at the completion of the study. A Monitoring Plan is established to outline roles and responsibilities.

## 14 Study governance

Phagenesis Ltd is the study Sponsor and has the overall responsibility for the conduct and safety of the study. The sponsor is responsible for assuring that the study meets the regulatory requirements of applicable competent authorities and governing institutional review boards/ethics committees. A Trial Steering Committee is established and is responsible for general oversight of the study. This committee will meet periodically to monitor clinical site progress and protocol compliance. The committee will be responsible for reviewing the final results. Clinical trial liability insurance is provided by the Sponsor for all study participants in accordance with applicable laws and regulations of the countries where the trial will be conducted to provide financial compensation in case of harm by study measures, if that should occur.

## 15 Safety oversight

A Medical Monitor (MM), in conjunction with the sponsor, will review adverse events (AE) of interest listings and will flag any events that require further review/investigation or if a safety review meeting is required. The MM is independent of Phagenesis and any study site. The MM's roles and responsibilities are outlined in a Safety Management Plan. An independent Data Monitoring Committee (DMC) will be established for the purposes of this study. The DMC will be responsible for monitoring aggregate safety information and the impact of AEs of interest on the safety and well-being of the patients, particularly if events relate to the Phagenyx system and study procedures. Safety and efficacy will be checked at the interim analysis. The DMC may recommend that the sponsor modify or stop the study based on safety information, or for efficacy/ futility. At the planned Interim Analysis, the DMC will also make recommendations on all pre-specified decision rules for study termination, continuation or adaption (e.g., Sample Size Re-estimation and/or population enrichment). The DMC members will be independent of Phagenesis Ltd and any participating study site. The DMC's roles and responsibilities will be outlined in a DMC Charter.

## 16 Protocol amendment(s)

Any revision/amendment(s) to the Protocol or Informed Consent documents will be submitted to applicable local/national ethics committees/institutional review boards in accordance with applicable regulations. Approvals will be obtained prior to implementing Protocol revisions at the sites.

## 17 Statistical considerations

### 17.1 Statistical hypotheses

The primary effectiveness analysis (Finkelstein-Schoenfeld (FS)^[32]^ (win-ratio) statistic) is on the composite of two endpoints in the following hierarchy based on clinical priorities:

1. Swallowing safety based on worst PAS score in series of up to 4 boli using thin stimuli (water) for each patient as determined by a FEES assessment on day 2 (approx. 24–60 hours) after completion of the final PES treatment, converted to a trichotomized ordinal response of safe (PAS 1–3), penetration (PAS 4–5), or aspiration (PAS 6–8).
2. DOSS score determined by bedside assessment 7 ± 1 days after completion of the final PES treatment.

Using the FS approach, each treatment arm patient will be compared to each control arm patient and a winner/loser determined. Comparisons are made hierarchically as defined above so that if the treatment arm patient performs better than the control arm patient with respect to the first hierarchy level (PAS) outcome then the treatment arm patient gets a score of +1 (winner) otherwise a score of −1 (loser) is assigned. If the first hierarchy level comparison is tied then the comparison is done with respect to the second outcome (DOSS) and the same scoring procedure is followed. The FS-statistic is based on the mean of the scores obtained from all such comparisons. If P is the probability that a randomly picked treatment arm patient performs better than a randomly picked control arm patient, then the expected value of the FS-statistic is given by 2 (P – 0.5). The hypothesis of interest is a one-sided null: H_0_: *P* ≤ .5 vs the one-sided alternative H_1_: *P* > .5. PAS and DOSS scores are ordinal outcomes. For each pair of patients evaluated (a pair being composed of one treated patient and one control patient), a binary outcome is derived that indicates whether the treated patient or the control patient performs better.

### 17.2 Sample size determination

Sample size calculations were carried out using simulations and are adjusted for interim analysis. Assuming that 50% of control patients will move from PAS score of 6–8 at baseline to a PAS score of 1–5 post-intervention and the corresponding number of patients in the PES treatment group is 65% (15% effect-size) and that the PES treatment group will benefit with a mean increase of 0.7 points on the DOSS score, 100 patients per arm will be required to achieve a power of at least 90%. Thus N_planned_ = 200 is the minimum sample size (unless the study is stopped early for futility at the only interim analysis). To allow for adaptations in design with respect to sample size and population enrichment, a maximum (evaluable) sample of N_max_ = 360 is pre-specified. To account for a 10% lost-to-follow-up, a maximum of 400 patients will be recruited.

### 17.3 Population for analyses

- The Intent to Treat (ITT) Population will consist of all patients that were randomised, irrespective of their protocol adherence and continued participation in the study.
- The Per Protocol (PP) Population will consist of all randomised patients who completed the full PES or Sham treatment regimen according to their randomisation assignment and for whom the composite primary endpoint data are available.
- The Safety Population will consist of all patients that were enrolled in the study and underwent the screening FEES with or without subsequent placement of the PES Catheter.

### 17.4 Statistical analyses

Tabulations will be produced for appropriate demographic, baseline, effectiveness and safety parameters. For categorical variables, summary tabulations of the number and percentage within each category (with a category for missing data) of the parameter will be presented. For continuous variables, the mean, median, standard deviation, minimum and maximum values will be presented. Time to event data will be summarized using Kaplan–Meier methodology using 25th, 50th (median), and 75th percentiles with associated 2-sided 95% confidence intervals, as well as percent of censored observations. Formal statistical hypothesis testing will be conducted at the 1-sided, 0.025 level of significance. A Statistical Analysis Plan (SAP) will detail all statistical analyses planned for this study and will be established prior to the planned Interim Analysis.

## 18 Discussion

This study evaluates the effectiveness of PES treatment in reducing severity of unsafe swallowing in recently extubated ICU patients with oropharyngeal dysphagia.

Post-extubation ICU-acquired dysphagia represents a major therapeutic challenge without evidence-based treatment.^[44]^ PES stimulates afferent sensory feedback for the responsible motor-cortex, activates cortico-bulbar pathways, and increases swallow-related neurotransmitter concentration, all contributing to improved swallowing.^[1,29–31]^ Safety data on the use of PES in acute stroke patients has been provided.^[35]^ The PHAST-TRAC study^[26]^ demonstrated that PES improved swallowing in dysphagic tracheotomized stroke patients, enabling earlier decannulation compared to controls. Moreover, pre-publication data from the PHADER registry^[45]^ showed improved swallowing and shorter length of hospital stay in 50 ICU patients with neurogenic dysphagia treated with PES. Therefore, previous trials have focused mainly on patients after stroke, with outcomes related to removal or avoidance of tracheostomy. In contrast, the present trial includes a general population of critically ill patients, and will answer whether PES reduces severity of unsafe swallowing. The strengths of this trail are its size, measurements of multiple dysphagia scores, and the inclusion of a general ICU population. The following limitations to this study seem apparent. First, although most dysphagia scales are validated, they may be considered somewhat subjective to interpretation, however, all investigators are trained on the correct interpretation prior to study start. Second, some of the exclusion criteria may theoretically overlap or could be perceived as not mutually exclusive. Third, inclusion of patients might be limited by the required expertise needed for FEES and PES, especially during weekends. Fourth, some could consider the fact that PhINEST is an industry-sponsored trial as a limitation, however, adequate blinding of the Sponsor (blinded) and all respective site staff (e.g., FEES and other assessment investigators (blinded) vs PES treaters (unblinded)) is ensured, and all authors of the paper will have full access to the data at the end of the trial.

In conclusion, this trial will answer the question, whether PES can reduce dysphagia severity in a general ICU population with oropharyngeal dysphagia after extubation.

## Acknowledgments

The authors thank all study physicians, nurses, research nurses, data managers and statistical staff for their dedicated support of PhINEST.

## Author contributions

**Conceptualization:** Joerg C. Schefold, Patrick Zuercher, Stephan M. Jakob, Satish Mistry.

**Funding acquisition:** Joerg C. Schefold.

**Methodology:** Joerg C. Schefold, Minna Bäcklund, Tero Ala-Kokko, Patrick Zuercher, Rajat Mukherjee, Satish Mistry, Stephan A. Mayer, Rainer Dziewas, Jan Bakker, Stephan M. Jakob.

**Supervision:** Joerg C. Schefold, Jan Bakker, Satish Mistry, Rajat Mukherjee, Stephan M. Jakob.

**Writing:** Joerg C. Schefold, Patrick Zuercher, Satish Mistry.

**Writing – review & editing:** Joerg C. Schefold, Minna Bäcklund, Tero Ala-Kokko, Patrick Zuercher, Rajat Mukherjee, Satish Mistry, Stephan A. Mayer, Rainer Dziewas, Jan Bakker, Stephan M. Jakob.

Patrick Zuercher orcid: 0000-0003-0331-4577.
